# Case report: a novel frameshift mutation in the *mitochondrial cytochrome c oxidase II* gene causing mitochondrial disorder

**DOI:** 10.1186/s12883-017-0883-5

**Published:** 2017-05-18

**Authors:** Laura Kytövuori, Mikko Kärppä, Hannu Tuominen, Johanna Uusimaa, Markku Saari, Reetta Hinttala, Kari Majamaa

**Affiliations:** 1 Research Unit of Clinical Neuroscience, University of Oulu, P.O. Box 5000, FI-90014 Oulu, Finland; 2 Medical Research Center Oulu, Oulu University Hospital and University of Oulu, Oulu, Finland; 3 Department of Neurology, Oulu University Hospital, P.O. Box 20, OYS, FI-90029 Oulu, Finland; 4 Department of Pathology, Cancer and Translational Medicine Research Unit, University of Oulu and Department of Pathology, Oulu University Hospital, Oulu, Finland; 5 PEDEGO Research Unit, University of Oulu, P.O. Box 5000, FI-90014 Oulu, Finland; 6 Department of Children and Adolescents, Division of Pediatric Neurology, Oulu University Hospital, Oulu, Finland; 7 Biocenter Oulu, University of Oulu, P.O. Box 5000, FI-90014 Oulu, Finland; 8 Turku Centre for Biotechnology, Cell Imaging Core, University of Turku, FI-20520 Turku, Finland

**Keywords:** Neuromuscular disorders, Mitochondrial diseases, Cytochrome c oxidase deficiency, Case report

## Abstract

**Background:**

*Mitochondrial cytochrome c oxidase 2*, *MT-CO2*, encodes one of the three subunits, which form the catalytic core of cytochrome c oxidase (COX), complex IV. Mutations in *MT-CO2* are rare and the associated phenotypes are variable including nonsyndromic and syndromic forms of mitochondrial diseases.

**Case presentation:**

We describe a 30-year-old man with cognitive decline, epilepsy, psychosis, exercise intolerance, sensorineural hearing impairment, retinitis pigmentosa, cataract and lactic acidosis. COX-deficient fibers and ragged red fibers were abundant in the muscle. Sequencing of mitochondrial DNA (mtDNA) revealed a novel frameshift mutation m.8156delG that was predicted to cause altered C-terminal amino acid sequence and to lead to truncation of the COX subunit 2. The deletion was heteroplasmic being present in 26% of the mtDNA in blood, 33% in buccal mucosa and 95% in muscle. Deletion heteroplasmy correlated with COX-deficiency in muscle histochemistry. The mother and the siblings of the proband did not harbor the deletion.

**Conclusions:**

The clinical features and muscle histology of the proband suggested a mitochondrial disorder. The m.8156delG deletion is a new addition to the short list of pathogenic mutations in the mtDNA-encoded subunits of COX. This case illustrates the importance of mtDNA sequence analysis in patients with an evident mitochondrial disorder.

## Background

Complex IV (CIV) in the mitochondrial respiratory chain is composed of 13 subunits, three of which are encoded by genes in mitochondrial DNA (mtDNA). *Cytochrome c oxidase II, MT-CO2*, codes for structural subunit 2, which participates in the formation of the catalytic core of CIV. Rare mutations have been described in *MT-CO2* in patients with various phenotypes, such as severe lactic acidosis [1], MELAS (mitochondrial encephalomyopathy, lactic acidosis, and stroke-like episodes) [2], encephalomyopathy [3], or rhabdomyolysis [4]. The 10 nuclear genes encoding the remaining subunits are even more rarely mutated than the mitochondrial ones and only a handful of mutations have been described to date [5–10]. Most of the cases with complex IV deficiency harbor causative mutation in the genes encoding nonstructural factors [11, 12].

We describe a male patient with heteroplasmic de novo deletion, m.8156delG, in *MT-CO2* leading to truncation of subunit 2 in cytochrome c oxidase (COX). The patient had a clinically apparent mitochondrial disorder with prominent findings in muscle histology.

## Case presentation

The patient is a 30-year-old man with negative family history of neuromuscular disorders. Early childhood development was normal. Sensorineural hearing impairment with tinnitus and mild memory deficit were the first symptoms, which were noticed at the age of 12 years. Neuropsychological examination revealed a moderate cognitive decline at the age of 24 years. Retinitis pigmentosa was diagnosed at the age of 25 years and premature cataract at the age of 29 years. The bilateral cataracts were operated soon after the diagnosis. Generalized epilepsy and psychosis were diagnosed at the age of 29 years, but because of poor tolerance and compliance he does not use medications. Moreover, he has exercise intolerance and mild balance disorder. He lives alone with supervision.

Neurological examination at the age of 29 years revealed cognitive decline, clumsy movements, abnormal balance tests and impaired visual acuity. Hearing was impaired and he had hearing aid in both ears. Ophthalmoplegia was not found. Walking and muscle strength were normal. His height is 170 cm and weight 57 kg.

**Fig. 1:**
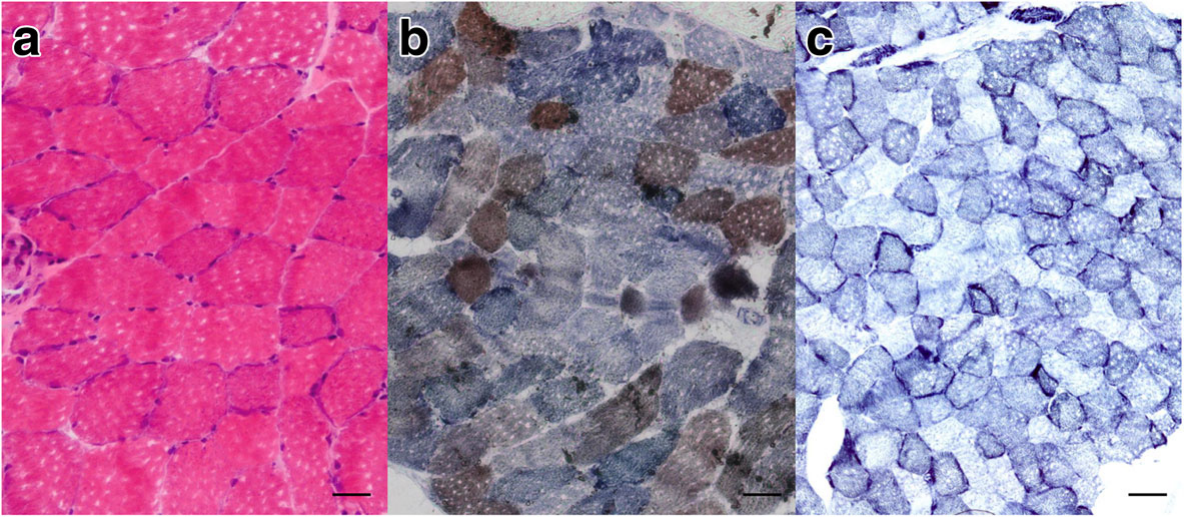
Histology of *vastus lateralis* muscle from the patient harboring the m.8156delG mutation. **a** Hematoxylin and eosin stainings show ragged *red fibers*. Bar = 50 μm (**b**) SDH-COX staining shows that COX-deficient fibers encompass more than half of the fibers. Bar = 50 μm (**c**) NADH staining shows mitochondrial proliferation. Bar = 100 μm

Blood lactate was first measured at the age of 23 years and was 3.06 mmol/l (laboratory reference, 0.33–1.33 mmol/l). Muscle histology at the age of 23 years revealed a substantial number of COX-deficient fibers and ragged red fibers (Fig. 1). Muscle ultrastructural analysis revealed large intramitochondrial inclusions. In brain MRI ventricular enlargement, moderate cerebellopontine atrophy and hyperintense signals in both basal ganglia were found. Cardiac examination and electrophysiological studies of the muscle and nerve were normal.

### Molecular methods and cell culture

DNA extraction from blood, buccal swab and myoblasts was done by using QIAamp DNA Blood Mini Kit (QIAGEN, Hilden, Germany). From muscle biopsy, the purification was done with Wizard® Genomic DNA purification kit (Promega Corporation, Madison, WI). The mitochondrial DNA was amplified and sequenced in 12 overlapping fragments.

Mutation heteroplasmy in tissues from the patient, his mother and the four siblings was determined by cloning as described previously [13]. One hundred colonies per cloned sample were screened for the mutation with the conformation sensitive gel electrophoresis.

A muscle sample was obtained from *vastus lateralis* and myoblast culture was established as described previously [14]. Cells were immunostained with myoblast-specific antibody for desmin (BioGenex, Fremont, CA, U.S.A.).

Laser-capture microdissection of COX-SDH stained frozen sections was done using Zeiss P.A.L.M. microscope (Microlaser Technologies GmbH, Bernried, Germany) in Turku Centre for Biotechnology. Ten COX-negative, COX-positive and COX-deficient fibers were collected in tubes with adhesive caps (Carl Zeiss MicroImaging GmbH, Munich, Germany). DNA was released from the ten pooled fibers of each COX phenotype using lysis buffer (200 mM KOH, 50 mM DTT) and 30 min incubation at 65 °C. The lysis reaction was then neutralized with Tris-HCl (900 mM, pH 8.3) and 4 μl of the solution was used for PCR. PCR was carried out using Phusion High-Fidelity DNA polymerase (Thermo Fisher Scientific, Waltham, MA, U.S.A.) in a 50 μl reaction that was established according to the provided protocol. The reaction conditions are available on request. The heteroplasmy was determined by cloning.

### Novel mutation in MT-CO2

**Fig. 2:**
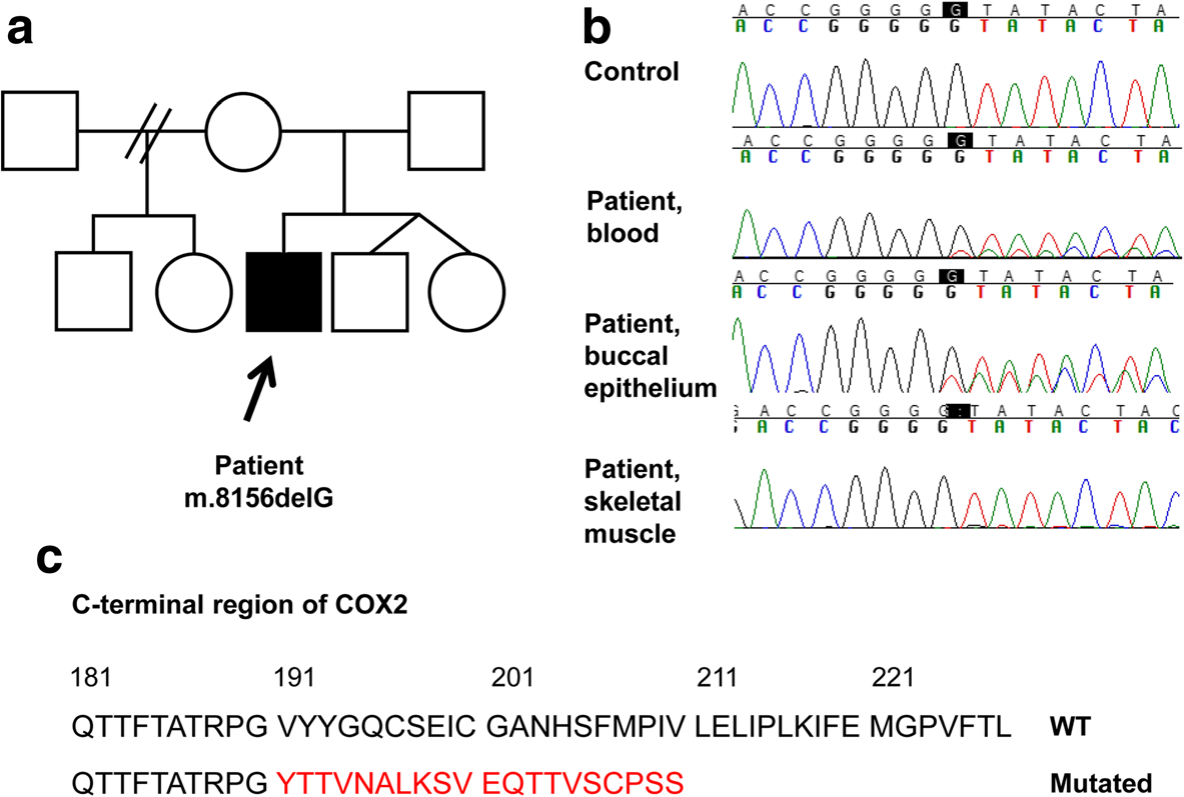
Novel frameshift mutation in *MT-CO2* alters and truncates the C-terminus of COX2. **a** The deletion m.8156delG was found in the proband, but not in the asymptomatic mother or siblings, suggesting that the mutation had arisen de novo. **b** The deletion was found in three tissues of the proband. **c** The mutation alters the amino acid sequence of the C-terminus of COX2 beyond amino acid 190 and creates a stop codon at position 211

Sequencing of mtDNA revealed a novel frameshift mutation m.8156delG in the *MT-CO2* gene of the proband (Fig. 2). The mutation was heteroplasmic and the proportion of the mutant genome was 26% in blood, 33% in epithelial cells from buccal swab and 95% in the skeletal muscle. The deletion was not detected in the blood or buccal mucosa from the mother or in the blood from the siblings of the patient suggesting de novo mutation. Single-fiber analysis showed that the proportion of the mutation required to cause a biochemical defect was high. Pooled blue COX-negative fibers contained 98% of mutant DNA. The proportion was 96% in COX-deficient fibers with intermediate color and 88% in biochemically normal fibers.

The mutation is predicted to lead to an altered amino acid sequence from position 191 and extending towards the C-terminus of the COX2 subunit. The mutation creates a termination codon resulting in premature truncation of the protein at amino acid 210, while the wild type protein consists of 227 amino acids. Three suggested copper binding sites are located in the affected region. The mtDNA belonged to haplogroup U8a1a1.

Four cell cultures were established from two muscle biopsies obtained on separate occasions. The first two cell cultures turned out to be fibroblasts with less than 20% of the mutant mtDNA. The other two cell cultures were desmin-positive verifying their muscular identity. Surprisingly, the mutation could not be detected in any of the subcultures.

## Discussion

We found a novel mutation m.8156delG that is suggested to result in an altered protein sequence and truncation of subunit COX2 in a patient with mitochondrial disorder. Previously, five mutations that are predicted to lead to truncated COX2 have been reported in patients with phenotypes of variable severity. In addition, six missense mutations in *MT-CO2* have been reported in patients with variable phenotypes in MITOMAP database [15]. Furthermore, nine variants have been reported with disease associations, but later studies have classified them as polymorphisms.

A frameshift deletion m.8042_8043delTA is one of the truncating mutations destroying one third of the COX2 and resulting in severe lactic acidosis and death at the age of few days [1]. The mutation load was 20% in the skeletal muscle and complex IV activity was decreased to half of the normal. The presence of the truncated form of COX2 was not verified in tissues, however. In contrast, the m.7630delT deletion has been found in a patient with MELAS syndrome that was fatal only at the age of 23 years [2]. The predicted length of COX2 was 16 amino acids, mutation heteroplasmy was 93%, and residual activity of CIV was only a few percent of the normal. Moreover, the protein level of COX2 was substantially reduced, but mRNAs of CIV subunits including that of mutant COX2 were significantly increased. The presence of the mutant protein was not investigated. Furthermore, a m.7896G > A nonsense mutation with a heteroplasmy of 60–80% has been reported to result in a 104-amino-acid protein, to lead to 13% activity of complex IV and to cause a severe early-onset multisystem disorder [16]. The amount of wild type COX2 was reported to be decreased, but the truncated protein was not detected. Finally, the mutation p.Glu129* has been found in a patient with adolescence-onset hearing impairment and psychiatric disorder. Mutation heteroplasmy was 90% in skeletal muscle and the remaining activity of complex IV was 12% [3]. Again, the mutant form of the protein was not investigated. At age 25 years he had developed bilateral cataract and within few years, the syndrome progressed to include gait instability, exercise intolerance and cardiac arrhythmia. The patient died from cardiac arrest at the age of 35 years.

These four cases show that in the presence of a truncating mutation the activity of CIV is decreased. Interestingly, there is a lack of correlation between decreased activity and clinical severity suggesting that the truncated proteins may interfere with respiratory chain or other cellular processes by gaining an abnormal function. Unfortunately, the studies have failed to show the presence of truncated COX2 or the existence has not been investigated.

The G stretch spanning between the nucleotides m.8152–8156 in *MT-CO2* has previously been found to be mutated in an adult patient with exercise-induced myoglobinuria and rhabdomyolysis [17]. This mutation was a heteroplasmic duplication m.8156dupG with a 56% mutation load in skeletal muscle causing decreased activity of CIV. In our patient, the predicted truncation of C-terminal sequence resulted in a mitochondrial disorder with various classical features of mitochondrial dysfunction. However, similar to the previous reports with frameshift mutations in *MT-CO2*, the presence of the truncated protein could not be verified.

The m.8156delG mutation was undetectable already in the first passages of the myoblast cultures despite of the high mutation load in the skeletal muscle showing that the mutation was present in highly differentiated myotubes. It is possible that the healthy cells benefitted from the culture conditions optimized for myoblasts leading to the disappearance of the mutant containing cells. Similar findings have previously been reported in a patient with m.9789 T > C in the *MT-CO3* with a heteroplasmy of 50% [3] and in a patient with m.9952G > A with a heteroplasmy of 57% [18]. Furthermore, m.7671 T > A in *MT-CO2* has been reported to be absent in myoblasts despite the very high heteroplasmy of 90% in the muscle [19]. In contrast, the m.6930G > A mutation in *MT-CO1* has been found at 33% heteroplasmy in myoblasts, while the mutation heteroplasmy was 75% in muscle [20]. Furthermore, heteroplasmic disease-causing deletions have been found to be present only in a minority of myoblast subcultures, even though the proportion of the deletion in the satellite cells was shown to be similar to that in the skeletal muscle [21]. In that study, the heteroplasmy decreased during further culturing and differentiation to myotubes. The authors suggested that there might be two periods of selection against the mutated mtDNA, the first occurring during satellite cell activation and second in the replication phase [21].

## Conclusions

Frameshift mutations in *MT-CO2* are rare and clinically variable. We presented here a patient with a progressive mitochondrial syndrome and a novel frameshift mutation m.8156delG in *MT-CO2* as a cause. This case illustrates the importance of mtDNA sequence analysis in patients with a suspected mitochondrial disorder.

## Abbreviations

mtDNA: mitochondrial DNA
*MT-CO2*: *mitochondrial cytochrome c oxidase 2*
COX: cytochrome c oxidase
MELAS: mitochondrial encephalomyopathy, lactic acidosis, and stroke-like episodes
